# Sodium Phenylbutyrate Ameliorates Ovariectomy-Induced Bone Loss in Rats

**DOI:** 10.3390/medicina61112016

**Published:** 2025-11-11

**Authors:** Bakiye Akbaş, Gülseren Dinç, Ahmet Akbaş, Nadir Adnan Hacım, Gülçin Ercan, Hatice Aygün, Oytun Erbaş

**Affiliations:** 1 Department of Obstetrics and Gynecology, Faculty of Medicine, Karadeniz Technical University, 61080 Trabzon, Turkey; bakiyeakbas@ktu.edu.tr (B.A.); gulserendinc1@gmail.com (G.D.); 2 Department of General Surgery, Faculty of Medicine, Karadeniz Technical University, 61080 Trabzon, Turkey; draakbas@hotmail.com; 3 Department of General Surgery, Güneşli Erdem Hospital, 34212 Istanbul, Turkey; adnanhcm@hotmail.com; 4 Department of General Surgery, Sultan 2. Abdulhamid Han Educational and Research Hospital, Istanbul Provincial Directorate of Health, 34668 Istanbul, Turkey; ghepgul@hotmail.com; 5 Neuroscience Laboratory, BAMER, Biruni University, 34015 Istanbul, Turkey; 6 Faculty of Medicine, BAMER, Biruni University, 34015 Istanbul, Turkey; oytunerbas2012@gmail.com

**Keywords:** ovariectomy, sodium phenylbutyrate, bone loss, oxidative stress, osteoporosis, cytokines

## Abstract

*Background and Objectives*: Estrogen deficiency after menopause accelerates bone loss through oxidative stress and inflammatory cytokines. Sodium phenylbutyrate (SP), a histone deacetylase inhibitor, exhibits antioxidative and anti-inflammatory effects, but its impact on postmenopausal osteoporosis remains unclear. *Materials and Methods:* Thirty female Wistar rats were divided into control, ovariectomy (OVX), and OVX+SPB groups (n = 10 each). After 12 weeks, bone mineral density (BMD), histomorphometry, bone marrow biomarkers (MDA, TNF-α, IL-6, RANKL), and plasma Cathepsin K were evaluated. *Results:* OVX induced trabecular deterioration with reduced number, area, and thickness (all *p* < 0.001), increased separation (*p* < 0.001), and decreased femoral and lumbar BMD (*p* < 0.001). SPB significantly improved these indices (TN, *p* < 0.05; TA, *p* < 0.01; TH, *p* < 0.05; femoral BMD, *p* < 0.05; lumbar BMD, *p* < 0.001; TS, *p* = 0.001). OVX elevated MDA, TNF-α, IL-6, RANKL, and Cathepsin K (all *p* < 0.001), which were significantly reduced by SPB (MDA, *p* < 0.001; TNF-α, *p* < 0.01; IL-6, *p* < 0.01; RANKL, *p* < 0.001; Cathepsin K, *p* < 0.001). *Conclusions:* SPB mitigates OVX-induced oxidative stress, inflammatory cytokine release, and osteoclast-mediated resorption, resulting in partial but significant improvements across biochemical, structural, and histomorphometric parameters in estrogen-deficient rats. Given its established clinical safety profile, SPB emerges as a cost-effective candidate for repurposing in postmenopausal osteoporosis, warranting further translational and clinical studies.

## 1. Introduction

Osteoporosis is a systemic skeletal disorder marked by low bone mass and microarchitectural deterioration, predisposing affected individuals to fragility fractures [1]. Postmenopausal osteoporosis, driven by estrogen deficiency, represents a major global burden, affecting >200 million individuals and causing ~9 million fractures annually [1,2]. Lifetime risk in women over 50 approaches one-third to one-half—about twice that of men—with hip, vertebral, and wrist fractures contributing substantially to morbidity, mortality, and costs [3,4,5,6].

Current therapies mainly suppress resorption (bisphosphonates, denosumab) but do not restore trabeculae, so absolute fracture risk reductions remain modest [7,8]. Anabolic agents (e.g., teriparatide) promote formation but are restricted by cost, daily injections, limited duration, and safety concerns. Long-term antiresorptives rarely cause serious adverse events yet fail to address impaired defect healing [9,10,11].

Postmenopausal osteoporosis is characterized by accelerated bone resorption driven by estrogen deficiency-induced osteoclast activation [12]. To replicate this pathophysiology, bilateral ovariectomy (OVX) in rats is widely used and endorsed by the FDA as a translational model of estrogen-deficiency-related bone loss [13,14]. Selective estrogen receptor modulators (SERMs), such as raloxifene and bazedoxifene, act as estrogen agonists in bone, suppressing osteoclastic activity and preserving bone mineral density [15,16]. SERM therapy has been shown to prevent bone loss and improve vertebral mechanical strength in OVX rats [17,18], while clinical trials have demonstrated reduction in vertebral fracture risk in postmenopausal women [19,20]. The ovariectomy (OVX) model, which mimics postmenopausal estrogen deficiency, demonstrates that estrogen loss creates a pro-oxidant, pro-inflammatory marrow milieu, with elevated ROS and cytokines (TNF-α, IL-1β, IL-6) that drive RANKL-coupled osteoclastogenesis while suppressing osteoblast survival, yielding net bone loss [21,22,23]. Thus, targeting oxidative and inflammatory pathways is a rational strategy for bone preservation and repair.

Recent evidence suggests that histone deacetylase inhibitors (HDACis) can significantly modulate estrogen receptor (ER) signaling in breast cancer. In ER-positive models, HDAC overexpression suppresses ESR1 (ERα) transcription, while HDAC inhibition reverses this effect, indicating that HDAC activity negatively regulates ER expression [24]. Multiple preclinical studies have further confirmed that HDACi treatment downregulates ESR1 expression and suppresses ER-responsive gene activity, thereby exerting an anti-estrogenic effect in hormone receptor-positive breast cancer [25,26,27,28]. Conversely, in ER-negative tumor models, certain HDAC inhibitors can restore silenced ER expression and re-sensitize tumors to anti-estrogen therapies such as tamoxifen [29,30]. Additionally, the class I-selective HDAC inhibitor entinostat has been shown to enhance endocrine responsiveness and improve aromatase inhibitor efficacy in hormone receptor-positive advanced breast cancer [31]. Together, these findings highlight the therapeutic relevance of the HDAC–ER axis in overcoming endocrine resistance in HR(+) breast cancer.

Epigenetic modulation via histone deacetylase (HDAC) inhibition has emerged as a dual-action approach. Excess HDAC activity impairs osteoblastogenesis and enhances osteoclast function, whereas HDAC inhibitors (HDIs) activate Runx2, increase mineralization, and suppress osteoclast differentiation partly through NF-κB/MAPK downregulation [32,33]. In ovariectomized models, pan-HDAC inhibition limits bone loss [34].

Sodium phenylbutyrate (SPB), an orally available class I HDAC inhibitor in clinical use, exerts strong anti-inflammatory and antioxidative effects by dampening NF-κB activation, lowering TNF-α/IL-1β/IL-6, suppressing iNOS/NO, enhancing glutathione, and reducing ROS [35,36]. These properties suggest that SPB could counteract postmenopausal bone loss while fostering regeneration.

SPB has shown potential in bone research, though data are scarce. Its active form, 4-phenylbutyric acid, protected against inflammatory bone loss by inhibiting osteoclast activity [37] and reduced glucocorticoid-induced osteoblast apoptosis [38]. Phenylbutyrate also prevented periodontal bone loss [39]. Moreover, sodium butyrate enhanced bone antioxidant capacity through GSK-3β/Nrf2 signaling [40]. To our knowledge, this is the first study to investigate the therapeutic potential of SPB in a postmenopausal osteoporosis model.

Here, we test whether SPB improves skeletal outcomes in an ovariectomized (OVX) rat model. We hypothesize that SPB, via anti-inflammatory and antioxidative mechanisms, mitigates OVX-induced trabecular and BMD deterioration and reduces marrow stress markers. By integrating histomorphometry, DEXA-based BMD, and bone-marrow biomarkers, this study explores SPB repurposing as a cost-effective adjunct for postmenopausal osteoporosis.

## 2. Material and Method

### 2.1. Animals

A total of 30 mature female Wistar albino rats (200–250 g) were used in this study. All experimental procedures were conducted in accordance with the National Institutes of Health (NIH, USA) Guide for the Care and Use of Laboratory Animals and were approved by the Institutional Animal Ethics Committee (Experimental Research Institute, Approval No: 7581526022, 18 December 2023). The animals were obtained from the Experimental Animal Laboratory of Science University. Rats were housed in pairs in stainless steel cages under controlled environmental conditions (22 ± 2 °C, 12 h light/dark cycle) with free access to standard chow and water ad libitum. Rats were provided the same standard laboratory chow from a single batch under identical housing conditions to minimize metabolic variation. Food intake was monitored weekly at the cage level.

#### Drug

Sodium phenylbutyrate (≥98% purity by HPLC; Sigma-Aldrich, St. Louis, MO, USA) was used in this study. The compound (C_10_H_11_NaO_2_; MW: 186.18 g/mol) is a white to off-white powder that is water-soluble (≥5 mg/mL at room temperature). For experimental use, the compound was dissolved in saline and administered by oral gavage at a dose of 1 mL/kg/day.

A daily dose of 200 mg/kg SPB was chosen based on previous preclinical studies showing its efficacy and safety in different animal models. This dose reduced neuroinflammation and oxidative stress in Parkinson’s and Alzheimer’s models [35,41] and attenuated hepatic ischemia–reperfusion injury by decreasing oxidative damage [42]. Comparable doses also alleviated inflammatory bone loss [43]. These findings collectively justify the use of this dose to evaluate SPB’s pharmacological potential in our OVX model.

### 2.2. Experimental Protocol

#### 2.2.1. Ovariectomy Procedure

Thirty adult female Wistar albino rats were used in this study. Twenty animals underwent bilateral dorsal ovariectomy, while ten rats were maintained as non-ovariectomized controls. For surgical procedures, anesthesia was induced via intraperitoneal injection of ketamine hydrochloride (50 mg/kg; Ketasol, Richterpharma AG, Wels, Austria) combined with xylazine hydrochloride (10 mg/kg; Rompun, Bayer, Leverkusen, Germany). Postoperatively, rats were allowed to recover and maintained for three weeks to establish the postmenopausal condition.

#### 2.2.2. Experimental Groups and Treatments

Following the recovery period, animals were randomly allocated into three experimental groups (n = 10 per group):

Group 1: Normal Control—non-ovariectomized rats receiving no treatment.

Group 2: OVX—ovariectomized rats receiving saline (1 mL/kg/day, oral gavage).

**Figure 1:**
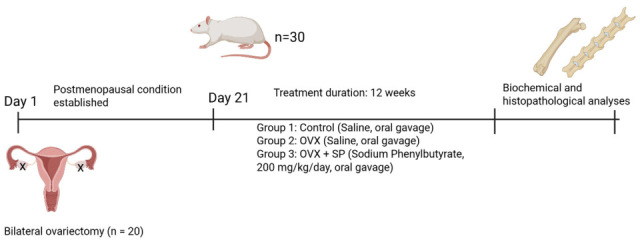
Experimental timeline.

Group 3: OVX + Sodium Phenylbutyrate—ovariectomized rats treated with sodium phenylbutyrate (200 mg/kg/day, oral gavage). (Figure 1).

All treatments were continued for 12 weeks.

#### 2.2.3. Bone Mineral Density (BMD) Measurements

**Figure 2:**
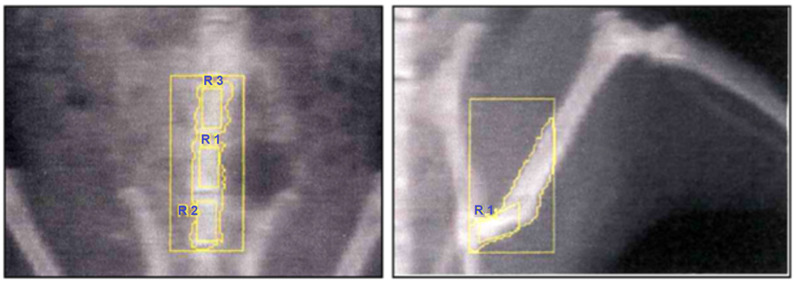
Representative DEXA scans of rat skeleton. (**Left**) Lumbar vertebrae with designated regions of interest (R1–R3) for BMD measurement. (**Right**) Femoral region with the selected ROI for femoral BMD determination.

At the end of the treatment period, bone mineral density (BMD) was measured under ketamine anesthesia (50 mg/kg, i.p.). Dual-energy X-ray absorptiometry (DEXA; Hologic QDR-4500A, Waltham, MA, USA) equipped with a small-animal software program was used. High-resolution scans were performed at two anatomical sites: the proximal femoral diaphysis of the left hind limb and the lumbar vertebrae (Figure 2).

##### Histomorphometric Evaluations

All histomorphometric evaluations were performed by two independent observers who were blinded to the group assignments. Inter-observer and intra-observer variability were assessed by repeating the measurements on randomly selected sections after a 2-week interval, and the consistency was confirmed using intraclass correlation coefficients (ICC > 0.85), indicating high reproducibility. Image quantification was conducted semi-automatically using the UTHSCSA Image Tool software (version 1.28, University of Texas Health Science Center, San Antonio, TX, USA), which minimizes user bias while allowing manual correction when required to ensure consistent measurement reproducibility.

#### 2.2.4. Sample Collection and Tissue Processing

Following BMD measurements, animals were sacrificed under deep anesthesia with ketamine (100 mg/kg, i.p.) and xylazine (50 mg/kg, i.p.) by cervical dislocation. Blood samples were obtained via cardiac puncture for biochemical analyses. Both femurs were excised and processed for histopathological and biochemical evaluations.

### 2.3. Histopathological Examination of Bone Tissue

#### 2.3.1. Tissue Preparation

For histological and morphometric evaluation, animals were anesthetized with an intraperitoneal injection of ketamine (40 mg/kg; Alfamine^®^, Ege Vet, Alfasan International B.V., Woerden, The Netherlands) and xylazine (4 mg/kg; Alfazyne^®^, Ege Vet, Alfasan International B.V., Woerden, The Netherlands), followed by transcardial perfusion with 200 mL of 4% formaldehyde in 0.1 M phosphate-buffered saline (PBS). The left femurs were carefully dissected, fixed in 10% neutral buffered formalin for 24 h at room temperature, and subsequently decalcified in 10% formic acid for 28 days. Following decalcification, tissues were processed routinely and embedded in paraffin. Transverse sections of 3 μm thickness were obtained using a Leica MR 2145 rotary microtome. Hematoxylin–eosin (H&E) staining was performed for histomorphometric analyses.

#### 2.3.2. Morphometric Analysis

For each specimen, five serial sections were collected from the proximal femoral metaphysis of the left hind limb. H&E-stained sections were examined under an Olympus light microscope at 20× magnification, and digital images were captured. Morphometric parameters were quantified using a semi-automated image analysis system (UTHSCSA Image Tool for Windows, Version 1.28). The following indices were evaluated: cortical thickness, trabecular number, trabecular thickness, trabecular area, and trabecular separation. Measurements were performed within 0.46 mm distal to the epiphyseal plate at equal distances from both cortical margins, in accordance with the methodology described by Parfitt et al. [44].

##### Evaluation of Histopathological Findings

The histopathological evaluation was performed using a semi-quantitative scoring system by two independent, blinded histologists. Inter-observer agreement was assessed using Cohen’s kappa coefficient (κ = 0.89)

#### 2.3.3. Measurement Criteria

Trabecular thickness was assessed by performing at least 50 measurements per trabecula, and data collection was continued until mean values stabilized. Trabecular number was determined by counting all trabeculae arranged in parallel within the defined metaphyseal region, located 0.46 mm distal to the epiphyseal plate. Trabecular area was calculated by delineating the trabecular boundaries in the same region where trabecular counts were performed. Cortical thickness was quantified as the mean of 50 independent measurements obtained from 3-μm histological sections. In addition, osteoblasts and osteoclasts were identified and enumerated in H&E-stained sections at 40× magnification, within 0.5 mm beneath the epiphyseal plate, using a digital image analysis program.

### 2.4. Inflammatory-Related Cytokine Analysis (IL-6, TNF-α, RANKL)

#### 2.4.1. Sample Preparation

Following euthanasia, femurs were rapidly excised and stored at −20 °C until analysis. Bone marrow was flushed from each femur with 1 mL isotonic saline, and the recovered material was homogenized in five volumes of phosphate-buffered saline (PBS; pH 7.4) using a glass homogenizer. Homogenates were centrifuged at 5000× *g* for 15 min, and the supernatants were collected for assays. Total protein concentration in the supernatants was determined by the Bradford method using bovine serum albumin as the standard.

#### 2.4.2. ELISA Quantification

Interleukin-6 (IL-6), tumor necrosis factor-α (TNF-α), and receptor activator of nuclear factor-κB ligand (RANKL) levels in bone marrow supernatants were measured using commercially available rat ELISA kits, strictly following the manufacturers’ instructions. All samples were assayed in duplicate. Absorbance was read on a microplate reader (Multiscan GO, Thermo Fisher Scientific, Waltham, MA, USA), and concentrations were calculated from standard curves and normalized to total protein content where applicable.

IL-6 was measured using the Rat IL-6 ELISA Kit (MyBioSource, San Diego, CA, USA, Cat. No. MBS2020158); TNF-α was quantified with the Rat TNF-α ELISA Kit (CUSABIO, Houston, TX, USA, Cat. No. CSB-E11987r-IS); and RANKL levels were determined using the Rat RANKL ELISA Kit (MyBioSource, Cat. No. MBS2022886). To ensure assay reliability beyond the manufacturers’ protocols, all samples were assayed in duplicate, and intra- and inter-assay coefficients of variation were maintained below 10% and 15%, respectively.

### 2.5. Measurement of Plasma Cathepsin K Levels

At termination, blood was drawn by cardiac puncture into EDTA tubes (1.5–2.0 mg/mL), processed on ice ≤ 30 min, and centrifuged (1500–2000× *g*, 10–15 min, 4 °C); plasma was clarified if needed (10,000× *g*, 5 min), aliquoted (≥100 µL), stored at −80 °C, and hemolyzed samples were excluded. Plasma Cathepsin K was analyzed using a Rat Cathepsin K (Cath-K) ELISA Kit (Sinogeneclon, Hangzhou, China, Cat. No. SG-20891) according to the manufacturer’s instructions. Pilot dilution tests ensured assay linearity (typically 1:2–1:4). Samples, standards, and controls were run in duplicate in a randomized layout, and absorbance was measured at 450 nm with a reference wavelength of 570–620 nm (Multiscan GO, Thermo Fisher Scientific). Concentrations were obtained from 4-parameter logistic curves, reported in pmol/L, and out-of-range samples were re-assayed after dilution. Quality was monitored with kit and in-house controls (intra/inter-assay CVs < 10%/<15%); plates outside control ranges were repeated, and pre-specified technical outliers were excluded before unblinding.

### 2.6. Measurement of Malondialdehyde (MDA)

MDA levels in bone marrow homogenates were determined by the thiobarbituric acid reactive substances (TBARS) assay. Briefly, samples were mixed with thiobarbituric acid (TBA) reagent and heated in a boiling water bath for 15 min. After cooling, the absorbance of the supernatant was measured at 532 nm using a spectrophotometer. Results were expressed as nmol MDA per mg protein, based on a standard curve constructed with 1,1,3,3-tetramethoxypropane [45].

### 2.7. Statistical Analysis

All data were inspected for distributional assumptions before hypothesis testing. Normality was evaluated with the Shapiro–Wilk test, and homogeneity of variances with Levene’s test. Given three independent groups (Control, OVX, OVX + Sodium phenylbutyrate), between-group comparisons for continuous outcomes (histomorphometric indices and biochemical measures) were performed using one-way ANOVA. When the homogeneity assumption held, pairwise comparisons were conducted with Tukey’s HSD; for variables violating homogeneity (e.g., lumbar BMD), Tamhane’s T2 was used. Results are reported as mean ± SEM. Exact two-tailed *p*-values are provided, with *p* < 0.05 considered statistically significant; very small probabilities are truncated to *p* < 0.001. Analyses were run in IBM SPSS Statistics (v26), and figures display mean ± SEM. 

#### Sample Size Determination and Power Analysis

The sample size in this study was determined based on comparable experimental designs reported in previous studies investigating the effects of natural or antioxidative compounds on bone loss in ovariectomized rat models [46,47,48]. These studies applied similar methodologies to assess bone mineral density and trabecular microarchitecture via DEXA or micro-CT and typically included 8–10 animals per group. Following this precedent and considering a 12-week treatment period with potential biological variability or attrition, ten rats were included in each experimental group.

A comprehensive statistical power analysis was subsequently conducted using G*Power version 3.1.9.7 (Heinrich-Heine-Universität Düsseldorf, Düsseldorf, Germany) to verify the adequacy of the experimental design and sample size. All analyses were performed using a one-way ANOVA (fixed effects, omnibus) with an α-error probability of 0.05 and a desired statistical power (1–β) of 0.95. The calculated effect sizes for morphometric parameters (trabecular number, area, separation, and thickness), bone mineral density (femoral and lumbar), and biochemical/inflammatory markers (MDA, TNF-α, IL-6, RANKL, and Cathepsin-K) ranged between f = 0.87–2.02, representing large to very large effects according to Cohen’s classification. The achieved power values (0.95–0.98) confirmed that the inclusion of 10 rats per group was sufficient to detect significant biological differences with high confidence and methodological reliability.

## 3. Results

### 3.1. Bone Histomorphometry and Bone Mineral Density

Assumption testing indicated that all parameters satisfied the normality assumption (Shapiro–Wilk, *p* > 0.05). Homogeneity of variances was confirmed by Levene’s test (*p* > 0.05) for all variables except lumbar BMD (*p* = 0.046). Therefore, one-way ANOVA was applied for group comparisons, followed by Tukey’s post hoc test for parameters with equal variances, while Tamhane’s T2 test was used for lumbar BMD due to heterogeneity.

**Figure 3:**
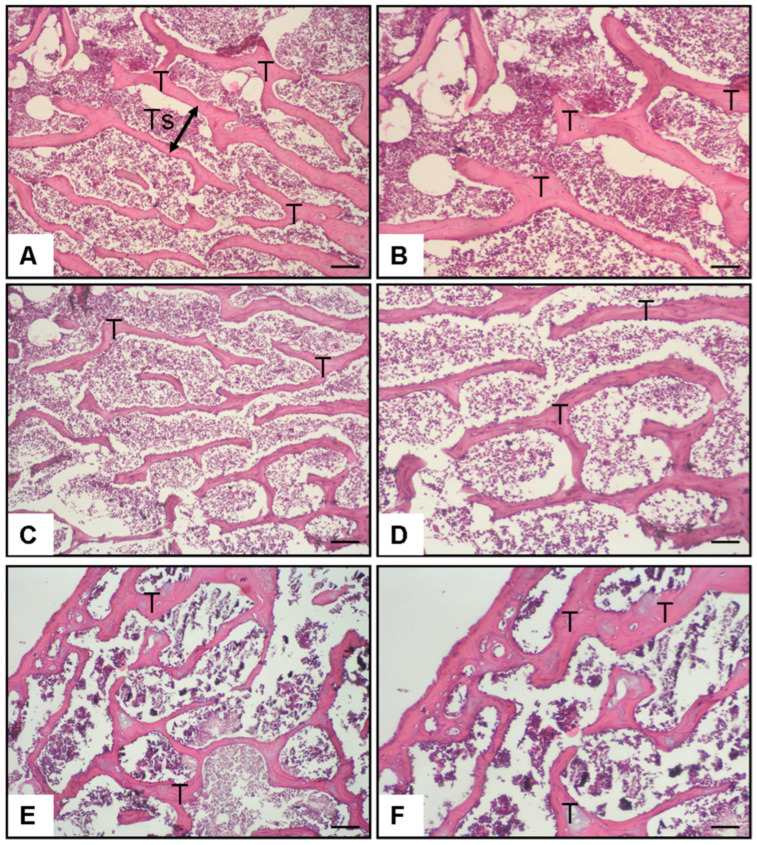
Representative photomicrographs of rat femoral trabeculae stained with H&E (20×). (**A**,**B**) Normal control group showing normal trabecular structure (T) and regular trabecular separation (Ts). (**C**,**D**) Ovariectomy group (12 weeks post-OVX) exhibiting trabecular thinning and widening of trabecular spaces. (**E**,**F**) Ovariectomy + sodium phenylbutyrate group demonstrating partial restoration of trabecular thickness (T) and improved trabecular architecture compared with OVX. The arrow indicates the Ts (intertrabecular space). Scale bar = 50 µm.

**Figure 4:**
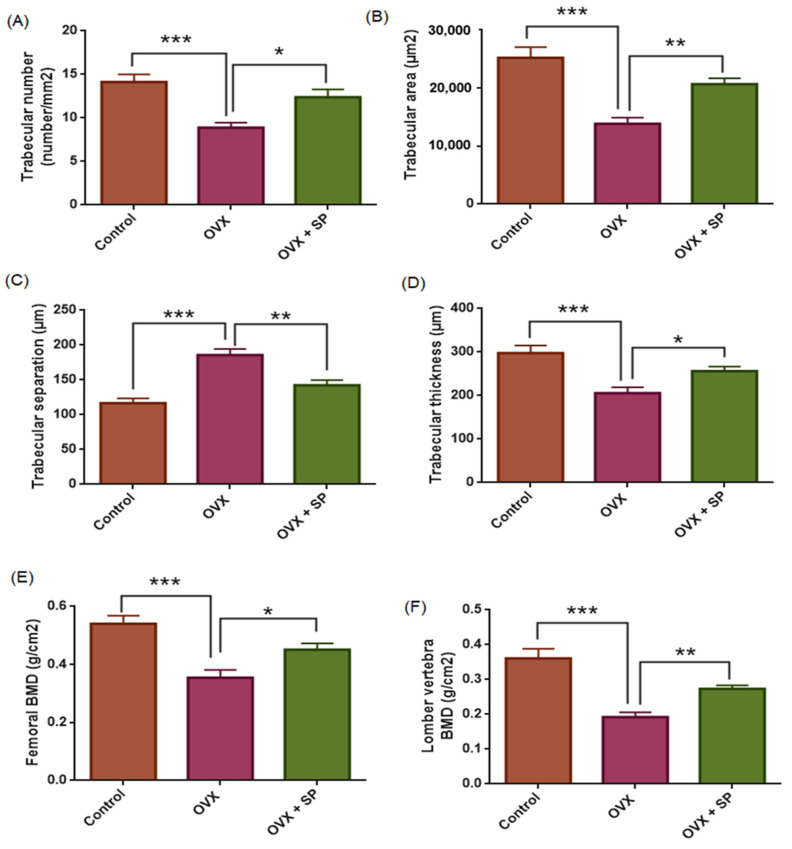
Effects of ovariectomy and sodium phenylbutyrate (SP) treatment on trabecular bone microarchitecture and bone mineral density (BMD). Trabecular number ((**A**), number/mm^2^), trabecular area ((**B**), µm^2^), trabecular separation ((**C**), µm), trabecular thickness ((**D**), µm), femoral BMD ((**E**), g/cm^2^), and lumbar vertebra BMD ((**F**), g/cm^2^) were measured in control, OVX, and OVX+SP groups. Data are presented as mean ± SEM (n = 10 per group). Statistical analysis was performed by one-way ANOVA followed by Tukey’s or Tamhane’s post hoc test where appropriate. * *p* < 0.05, ** *p* < 0.01, *** *p* < 0.001.

Histomorphometric analysis revealed marked group differences in trabecular bone architecture and BMD. Relative to the control group (Figure 3A,B and Figure 4), ovariectomized rats (Figure 3C,D and Figure 4), showed a pronounced deterioration in bone microstructure, characterized by significant reductions in trabecular number (TN; *p* < 0.001), trabecular area (TA; *p* < 0.001), and trabecular thickness (TH; *p* < 0.001), together with a significant increase in trabecular separation (TS; *p* < 0.001) (Figure 4). Consistently, both femoral and lumbar BMD values were markedly decreased in the OVX group compared with controls (*p* < 0.001).

Treatment with sodium phenylbutyrate partially reversed these alterations. Compared with the OVX group, sodium phenylbutyrate significantly increased TN (*p* = 0.01), TA (*p* = 0.004), TT (*p* = 0.041), and BMD (femoral, *p* = 0.032; lumbar, *p* = 0.001), while reducing TS (*p* = 0.001) (Figure 3E,F and Figure 4). However, the improvements did not fully restore the parameters to control levels. These results confirm the successful establishment of the estrogen deficiency osteoporosis model and demonstrate that sodium phenylbutyrate ameliorates trabecular bone deterioration.

### 3.2. Oxidative Stress Marker (MDA)

Assumption testing indicated that bone marrow MDA values met the normality assumption (Shapiro–Wilk, *p* > 0.05) and the homogeneity of variances (Levene’s test, *p* = 0.714). Therefore, one-way ANOVA was applied, followed by Tukey’s post hoc test.

**Figure 5:**
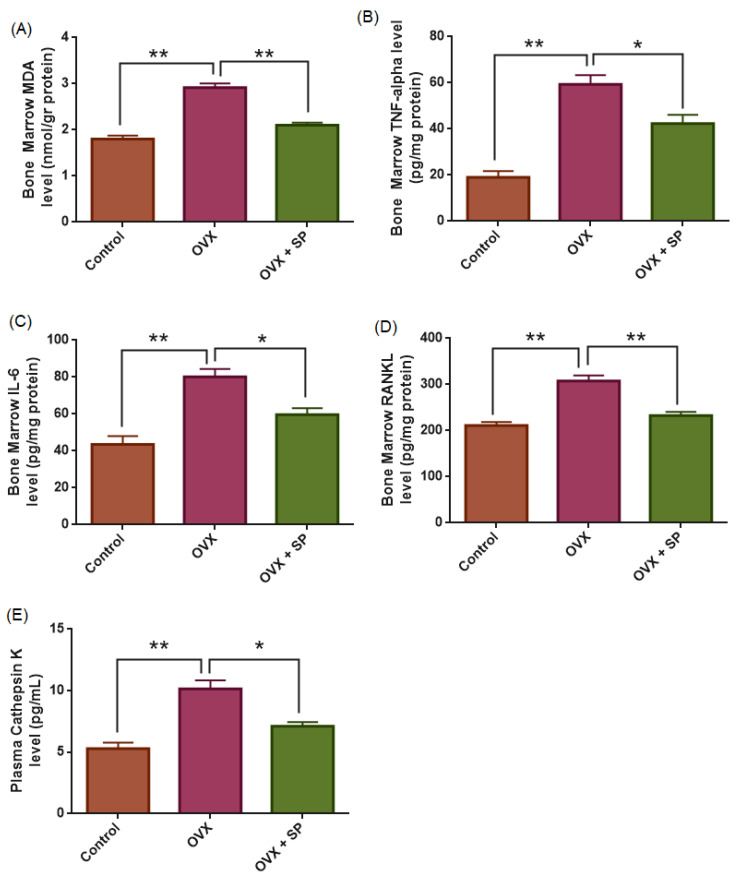
Effects of ovariectomy and sodium phenylbutyrate (SP) treatment on bone marrow oxidative stress, inflammatory cytokines, RANKL, and plasma Cathepsin K levels. Bone marrow MDA ((**A**), nmol/g protein), TNF-α ((**B**), pg/mg protein), IL-6 ((**C**), pg/mg protein), and RANKL ((**D**), pg/mg protein) as well as plasma Cathepsin K ((**E**), pmol/L) were measured in control, OVX, and OVX + SP groups. Data are expressed as mean ± SEM (n = 10 per group). Statistical analysis was performed using one-way ANOVA followed by Tukey’s post hoc test. * *p* < 0.05, ** *p* < 0.001.

ANOVA revealed a highly significant group effect for bone marrow MDA levels (F(2,27) = 59.799, *p* < 0.001). Compared with the control group (1.8 ± 0.07 nmol/g protein), ovariectomized rats displayed a marked elevation in MDA concentrations (2.9 ± 0.08 nmol/g protein; *p* < 0.001), indicating that ovariectomy induces oxidative stress in bone marrow. Administration of sodium phenylbutyrate significantly reduced MDA levels (2.10 ± 0.05 nmol/g protein) relative to the OVX group (*p* < 0.001), suggesting that sodium phenylbutyrate exerts a partial ameliorative effect against oxidative stress. However, MDA remained slightly higher compared with the control group (*p* = 0.022) (Figure 5).

### 3.3. Inflammatory Cytokines (TNF-α and IL-6)

Assumption testing indicated that bone marrow TNF-α and IL-6 values satisfied the normality assumption (Shapiro–Wilk, *p* > 0.05) and homogeneity of variances (Levene’s test, TNF-α: *p* = 0.333; IL-6: *p* = 0.503). Therefore, one-way ANOVA was performed, followed by Tukey’s post hoc test.

For TNF-α, ANOVA revealed a robust group effect (F(2,27) = 31.922, *p* < 0.001). Ovariectomized rats exhibited markedly elevated TNF-α levels (59.3 ± 4 pg/mg protein) compared with controls (19.0 ± 2.7 pg/mg protein; *p* < 0.001). Sodium phenylbutyrate administration significantly reduced TNF-α concentrations (42.2 ± 3.8 pg/mg protein) relative to the OVX group (*p* = 0.006), but levels remained higher than in controls (*p* < 0.001).

Similarly, IL-6 levels demonstrated a significant group difference (F(2,27) = 19.543, *p* < 0.001). Ovariectomy markedly increased IL-6 (80.1 ± 4.3 pg/mg protein) compared with controls (43.5 ± 4.5 pg/mg protein; *p* < 0.001). Sodium phenylbutyrate treatment significantly decreased IL-6 (60 ± 3.5 pg/mg protein) compared with OVX (*p* = 0.005), although values did not fully normalize to control levels (*p* = 0.028).

Based on our results, estrogen deficiency is associated with elevated pro-inflammatory cytokine levels in bone marrow, whereas sodium phenylbutyrate shows a partial anti-inflammatory response (Figure 5).

### 3.4. RANKL

Assumption testing indicated that bone marrow RANKL values satisfied the normality assumption (Shapiro–Wilk, *p* > 0.05) and homogeneity of variances (Levene’s test, *p* = 0.620). Therefore, one-way ANOVA with Tukey’s post hoc test was applied.

ANOVA demonstrated a significant group effect for RANKL levels (F(2,27) = 28.271, *p* < 0.001). Ovariectomized rats exhibited significantly higher RANKL concentrations (307.8 ± 12.0 pg/mg protein) compared with the control group (211.1 ± 7.7 pg/mg protein; *p* < 0.001), indicating enhanced osteoclastogenesis and bone resorption activity. Treatment with sodium phenylbutyrate reduced RANKL levels (232.7 ± 8.2 pg/mg protein), showing a significant decrease relative to the OVX group (*p* < 0.001), although values remained non-significantly higher than controls (*p* = 0.263). The decrease in RANKL levels indicates that sodium phenylbutyrate partially suppresses osteoclast differentiation and bone resorption, contributing to its ameliorative effect on bone integrity (Figure 5).

### 3.5. Plasma Cathepsin K

Assumption testing indicated that plasma Cathepsin K values satisfied the normality assumption (Shapiro–Wilk, *p* > 0.05) and homogeneity of variances (Levene’s test, *p* = 0.178). Accordingly, one-way ANOVA with Tukey’s post hoc test was performed.

A significant group effect was observed for Cathepsin K levels (F(2,27) = 20.993, *p* < 0.001). Ovariectomized rats exhibited markedly higher Cathepsin K concentrations (10.14 ± 0.70 pmol/L) compared with controls (5.28 ± 0.49 pmol/L; *p* < 0.001), reflecting enhanced osteoclastic activity. Administration of sodium phenylbutyrate significantly reduced Cathepsin K levels (7.10 ± 0.35 pmol/L) relative to the OVX group (*p* = 0.001), though concentrations remained slightly elevated compared with controls (*p* = 0.059). These findings indicate that sodium phenylbutyrate exerts a partial ameliorative effect (Figure 5).

### 3.6. General Observations

Throughout the experimental period, no adverse events, morbidity, or mortality were observed. All animals remained in good general condition and tolerated the experimental procedures well. According to the institutional animal ethics protocol, predefined humane endpoints included >15% body weight loss, impaired locomotion, inability to access food or water, and markedly reduced response to stimuli. None of these criteria were observed during the study. No significant differences in body weight were observed among the groups throughout the study period.

## 4. Discussion

This study is the first to demonstrate the protective effects of sodium phenylbutyrate against OVX-induced bone loss in an experimental osteoporosis model. Treatment improved trabecular microarchitecture and bone density, suppressed oxidative stress and marrow cytokines, and reduced RANKL and circulating Cathepsin K, indicating inhibition of osteoclast-mediated resorption. To our knowledge, this is the first evaluation of SPB in the OVX model with plasma Cathepsin K and bone marrow cytokines, highlighting its potential as a candidate therapy for estrogen-deficiency-related bone loss.

It is well established that estrogen deficiency accelerates skeletal deterioration, with trabecular bone being especially vulnerable [49,50]. Consistent with previous reports [12,51], our results showed significant reductions in trabecular number, thickness, and area, along with increased separation, confirming selective trabecular fragility. Histological evidence of trabecular thinning paralleled the densitometric decline in femoral and lumbar BMD, supporting the robustness of our model. While dual-energy X-ray absorptiometry (DEXA) remains the diagnostic gold standard, it does not fully capture fracture risk [52,53]. Incorporating trabecular microarchitecture therefore adds translational value for evaluating bone strength [54,55].

In this validated model, SPB treatment partially restored trabecular structure and bone density, improving trabecular number, thickness, and area compared with untreated OVX rats. These findings indicate reduced bone resorption and preserved microarchitecture. In clinical settings, denosumab and bisphosphonates increase spine BMD by 5–8% within 1–3 years [56], and teriparatide achieves 9–13% after 18–24 months [57]. Although SPB produced smaller absolute BMD gains than long-term human therapies, the 12-week treatment in rats corresponds to roughly a decade of human exposure, indicating sustained antiresorptive efficacy and translational promise for osteoporosis management.

A growing body of evidence indicates that oxidative and nitrosative stress jointly contribute to estrogen-deficiency-induced bone loss [58,59,60]. The rise in MDA reflects intensified ROS-driven lipid peroxidation within bone tissue. Estrogen withdrawal promotes ROS overproduction and pro-inflammatory cytokine release, shifting the bone microenvironment toward resorption dominance by stimulating osteoclastogenesis and impairing osteoblast differentiation [61,62]. Experimental studies in OVX rats consistently demonstrate increased ROS and MDA levels accompanied by diminished antioxidant enzyme activity, indicating a disrupted redox balance [63,64]. Previous reports also showed that antioxidant treatments such as vitamin C, strontium ranelate, or nitrate ameliorate bone loss by restoring redox homeostasis [12,65,66]. In line with these findings, our data revealed elevated bone marrow MDA in OVX rats, which was significantly attenuated by SPB, suggesting that SPB exerts an ameliorative effect against oxidative stress-mediated bone fragility. The decrease in MDA levels observed in SPB-treated OVX rats suggests restoration of redox homeostasis within the bone microenvironment.

RANKL and its receptor RANK, members of the TNF superfamily, are central drivers of osteoclast differentiation. Estrogen deficiency enhances inflammatory signaling, with TNF-α and IL-6 emerging as pivotal mediators of OVX-induced bone loss. Elevated TNF-α and IL-6 have been consistently reported in OVX rats [67,68,69], and TNF-overexpressing mice show severe trabecular deterioration associated with increased IL-1β and RANKL [70]. Mechanistically, TNF-α stimulates RANKL expression and amplifies RANKL-driven osteoclastogenesis through PI3K/Akt signaling, thereby accelerating bone resorption [23,71,72]. Under estrogen-deficient conditions, TNF-α-driven RANKL signaling promotes ROS generation, which in turn amplifies NF-κB/NFATc1 activation and osteoclast differentiation, thereby contributing to bone resorption [73,74]. Consistent with this evidence, our data revealed marked increases in TNF-α, IL-6, and RANKL in OVX rats, paralleling structural deterioration. Notably, sodium phenylbutyrate significantly reduced these cytokines, suggesting it mitigates estrogen-deficiency-induced inflammation and disrupts the TNF-α/IL-6–RANKL axis. This dual action on inflammatory mediators and osteoclastogenic signaling underscores its therapeutic promise in postmenopausal osteoporosis.

Cathepsin K is a recognized marker of bone resorption [75]. In ovariectomized (OVX) rats, enhanced osteoclast activity is consistently associated with elevated cathepsin K expression and accelerated bone loss [76,77]. Similarly, our data showed increased plasma cathepsin K in OVX rats compared with sham controls. Selective cathepsin K inhibition in OVX models has been reported to attenuate osteoclast-mediated resorption, improve BMD, and preserve microarchitecture, thereby providing antifracture efficacy [78,79,80,81]. Consistent with these findings, sodium phenylbutyrate prevented the rise in cathepsin K, indicating suppression of bone resorption and anti-osteoporotic potential.

The protective effects of sodium phenylbutyrate likely arise from its histone deacetylase (HDAC) inhibitory activity, which enhances Nrf2-driven antioxidant defenses while suppressing NF-κB-mediated cytokine transcription (TNF-α, IL-6) [35,36,82,83]. This mechanism explains the observed reductions in MDA, TNF-α, and IL-6, together with the attenuation of RANKL signaling and cathepsin K release, ultimately limiting osteoclast differentiation and bone resorption. Collectively, these findings provide novel evidence that sodium phenylbutyrate mitigates estrogen-deficiency-induced osteoporosis by reducing oxidative stress, inflammation, and osteoclast-mediated bone loss. Although its antiresorptive potency appears weaker than that of estrogen replacement, bisphosphonates, or denosumab, its distinct upstream mechanism highlights its potential as a complementary or safer alternative in postmenopausal osteoporosis management.

SPB shows cross-species efficacy, supporting its translational potential. Its analog 4-PBA improved bone parameters in rodents [37] and extended lifespan in *Drosophila* [84]. It also enhanced neurological outcomes in chronic mouse models [85]. Clinically, SPB is FDA-approved for urea cycle disorders, with proven long-term safety in children and adults [86]. In the present study, 12 weeks of SPB administration in rats—approximately equivalent to 10 human years—suggests favorable long-term safety and cost-effectiveness, supporting its potential as a promising therapeutic candidate for osteoporosis.

Structurally distinct from 17β-estradiol and SERMs, sodium phenylbutyrate (SPB) lacks affinity for estrogen receptors but can epigenetically influence ER-related signaling through HDAC inhibition. Prior studies show that HDAC inhibition alters ERα expression in breast and mammary models [25,87], while butyrate analogs activate ERα/AMPK signaling under estrogen-deficient conditions [88]. In our OVX rats, SPB improved bone density and trabecular structure while lowering TNF-α, IL-6, MDA, RANKL, and Cathepsin K, indicating anti-inflammatory and antioxidant effects. Thus, its skeletal protection likely stems from indirect epigenetic modulation of ER-dependent pathways rather than direct receptor binding in estrogen-deficient bone.

Although HDAC inhibitors exert broad epigenetic effects, their limited tissue selectivity may cause systemic toxicity. Pan-HDAC inhibitors such as vorinostat have been associated with fatigue, gastrointestinal disturbances, and cytopenia [89], while 4-phenylbutyrate worsened cardiac function in a heart failure mouse model [90]. Because HDACs regulate transcription in multiple organs, off-target effects involving neurocognitive, gastrointestinal, hematologic, or cardiovascular systems are possible. Careful dosing, chronic toxicological evaluation, and development of isoform-selective HDAC inhibitors are therefore critical for clinical translation.

Current osteoporosis therapies—including bisphosphonates, denosumab, and teriparatide—are effective but limited by adverse effects, high cost, and poor adherence. Bisphosphonates may cause gastrointestinal irritation and rare osteonecrosis of the jaw [91,92]; denosumab withdrawal can trigger rebound fractures [93]; and teriparatide use is restricted by cost, hypercalcemia, and treatment duration [57,94]. In contrast, SPB offers favorable tolerability and affordability, with preclinical and clinical data supporting its anti-inflammatory, antioxidant, and metabolic benefits [41,95].

SPB also shows a generally safe long-term profile, although mild adverse effects such as gastrointestinal symptoms, metabolic acidosis, electrolyte imbalance, and sodium-related edema or hypertension have been reported [86,96]. At higher doses, transient neurocognitive or metabolic symptoms such as sedation, confusion, or memory disturbance have been documented in oncology trials [97]. Rare events, including mild hepatic enzyme elevation or neuro-metabolic symptoms, may occur at higher doses [86,98]. Therefore, prolonged use should incorporate individualized dosing and periodic biochemical monitoring to minimize cumulative risk, particularly during chronic administration.

The dose used in this study (200 mg/kg/day) corresponds to a human-equivalent dose of approximately 32 mg/kg/day, well below the clinically approved range for urea cycle disorders (450–600 mg/kg/day, up to ~20 g/day in adults; PHEBURANE^®^ FDA label, [99]), supporting its feasibility for human use. However, the high sodium content and pill burden of SPB may limit long-term adherence in elderly patients; thus, sustained-release or low-sodium formulations should be prioritized in future translation.

SPB’s antiresorptive mechanism, consistent with NF-κB-regulated osteoclast inhibition [37], suggests limited additive benefit with other antiresorptive drugs such as bisphosphonates or denosumab [100], since agents acting through overlapping pathways may over-suppress bone remodeling [101]. Conversely, SPB’s antioxidative and osteoblast-protective actions could complement anabolic therapies like teriparatide (PTH 1–34) by mitigating oxidative-stress-driven resorption, as evidenced by the DATA-Switch trial [102], which showed enhanced BMD gains through sequential anabolic–antiresorptive therapy. Therefore, SPB may act synergistically with anabolic, but redundantly with antiresorptive, agents.

### Future Directions

We are currently extending this study to longer treatment durations to confirm the long-term efficacy and safety of SPB. Combination studies with standard antiresorptive (alendronate) and anabolic (teriparatide) agents are also planned to evaluate potential synergistic effects. Ongoing work includes biomechanical testing to assess bone strength and fracture resistance, along with chronic safety evaluations through serum and organ analyses. Future studies will also incorporate advanced bone analyses such as micro-CT and dynamic histomorphometry, as well as additional biochemical markers (e.g., P1NP, CTX) to better characterize SPB’s dual antiresorptive and anabolic potential. From a translational perspective, clinical development will likely follow a repurposing pathway, beginning with dose-finding and safety trials using optimized low-sodium or sustained-release SPB formulations, followed by larger studies to establish efficacy and obtain regulatory approval for osteoporosis therapy.

## 5. Conclusions

Sodium phenylbutyrate partially restored trabecular microarchitecture and bone density, reduced oxidative stress and pro-inflammatory cytokines, and suppressed RANKL and Cathepsin K in OVX rats. These results suggest that SPB mitigates estrogen-deficiency-induced bone fragility. While limited to an experimental model, our findings provide a rationale for further preclinical and clinical studies to establish its translational relevance in postmenopausal osteoporosis.

## 6. Limitation

This study has several limitations. First, micro-CT analysis was not performed, restricting bone evaluation to BMD and histological indices rather than three-dimensional parameters such as trabecular thickness and connectivity. In addition, only Cathepsin K was measured as a marker of osteoclast activity. Although other markers such as TRAP, OPG, MMP-9, ALP, osteocalcin, or Runx2 could better characterize bone remodeling, Cathepsin K was selected for its high specificity [103,104]. Serum bone turnover markers (P1NP, CTX), dynamic histomorphometry, and mechanistic assays for HDAC activity or histone acetylation were also not performed; therefore, the anabolic or antiresorptive nature of SPB’s effects and its epigenetic mechanism can only be inferred from the available data. Future studies should include serum bone turnover markers, dynamic histomorphometry, and HDAC activity analyses to elucidate these mechanisms more comprehensively.

Second, the treatment duration was limited to 12 weeks, preventing evaluation of long-term persistence or plateau effects. A post-treatment follow-up phase was also not included; thus, it remains uncertain whether SPB’s bone-protective effects would persist after withdrawal.

Third, only ovariectomized female rats were used. While this model reflects postmenopausal osteoporosis, sex-related differences in bone turnover and drug response are known [105,106]. Future studies should include male or orchidectomized models to clarify potential sex-dependent effects.

Fourth, non-skeletal tissues were not systematically evaluated. Although no overt toxicity was observed and animals remained healthy, future work should include serum biochemistry and histopathology of major organs to confirm systemic safety.

Finally, this preliminary study did not include a positive control group to benchmark SPB against standard therapeutic agents. This decision was made to minimize animal use in accordance with ethical guidelines, but future investigations will incorporate such comparators to better contextualize SPB’s efficacy.

## Data Availability

Data are available upon request from the corresponding author.

## Abbreviations

ROS—Reactive oxygen species; TNF-α—Tumor necrosis factor alpha; IL-6—Interleukin-6; RANKL—Receptor activator of nuclear factor-κB ligand; CTSK—Cathepsin K; SPB—Sodium phenylbutyrate; OB—Osteoblast; OC—Osteoclast; OVX—Ovariectomy.
